# Diethylcarbamazine elicits calcium signals by activation of Brugia malayi TRP-2b channels heterologously expressed in HEK293 cells

**DOI:** 10.21203/rs.3.rs-7359086/v1

**Published:** 2025-09-08

**Authors:** Paul D. E. Williams, Sudhanva S. Kashyap, Alan P. Robertson, Richard J. Martin

**Affiliations:** Iowa State University; Iowa State University; Iowa State University; Iowa State University

**Keywords:** Diethylcarbamazine, HEK293 cells, TRP-2, Brugia malayi, calcium imaging

## Abstract

Diethylcarbamazine is a classic anthelmintic that is used for the prevention and treatment of lymphatic filariasis. The mode of action of diethylcarbamazine is still not well understood with the consensus that it acts on the host immune system, rather than directly acting on the adult parasite. Recent studies, have found that diethylcarbamazine acts on the muscle of adult female *Brugia malayi*, generating temporary spastic paralysis mainly through the Transient Potential Receptor C (TRPC) orthologue TRP-2. Activation of TRP-2 leads to inward currents on the muscle, an increase in intracellular calcium and subsequent muscle contraction. These studies have demonstrated that *Brugia malayi* TRP-2 is activated by diethylcarbamazine. In this study, we heterologously expressed the *Brugia malayi* TRP-2b channel in the Human Embryonic Kidney (HEK) 293 cell line. Application of diethylcarbamazine to *Bma-trp-2b* transfected HEK293 cells leads to larger and more frequent increases in intracellular calcium compared to non-transfected cells. This increase can be inhibited using the TRPC specific antagonist SKF96365. Our study shows that diethylcarbamazine’s action is dependent upon the *Brugia malayi* TRP-2 channel and may also, in addition, activate endogenous mammalian TRP channels.

## Introduction

Lymphatic filariasis is a neglected tropical disease caused by the filarial parasites *Wuchereria bancrofti, Brugia malayi* and *Brugia timori* with over 657 million people worldwide threatened by the illness and 120 million people affected^1,2^. Parasites are transmitted between hosts through biting insects, and the adults live within the lymphatic vessels. In severe cases, parasites can block lymphatic ducts resulting in disfiguring visible manifestations including lymphoedema and elephantiasis. The swelling of limbs can prevent individuals from working and lead to societal rejection. There are no effective vaccines against filarial parasites, nor have the measures to control the spread by their vectors been sufficient.

Control of lymphatic filariasis relies on the use of chemotherapeutic agents that target microfilaria in the blood to reduce the risk of transmission between individuals through biting insects. Common compounds used to treat individuals include the benzimidazoles (albendazole) and macrocyclic lactones (ivermectin). These compounds can be administered alone or in combination through Mass Drug Administration (MDA) programs. Although these compounds are effective at killing and clearing the microfilariae, they are less effective against the adult parasites that can live in the host for 6–8 years. A third drugs, diethylcarbamazine is also used to treat lymphatic filariasis. However, it is not recommended in regions with high levels of onchocerciasis, the cause of river blindness, because of concerns over severe adverse reactions.

Previously, the mechanism of action for diethylcarbamazine was understood to be mediated by the host immune system that targeted and cleared the microfilaria from the blood^3–6^, rather than a direct effect on the adult parasite. However, recent studies have demonstrated that diethylcarbamazine does have a direct effect on adult *Brugia malayi* by causing temporary spastic paralysis^7^. The paralytic effect was identified to be mediated by Transient Receptor Potential (TRP) channels, primarily the TRPC orthologue TRP-2, and a smaller part through the TRPM channel orthologues, GON-2 and CED-11^7,8^. Paralysis is caused by diethylcarbamazine acting on the parasite body muscles cells, generating an inward depolarizing current through opening the TRP-2 channels, entry of calcium which in turn activates the calcium-dependent SLO-1 K channel^7,8^. Interestingly, the co-application of diethylcarbamazine and the SLO-1 targeting anthelmintic emodepside^9–11^ has a potentiating effect, resulting in stronger calcium signals and stronger paralytic effects compared to using either compound alone^8,12^. The activation of TRP channels by diethylcarbamazine is not limited to *Brugia malayi* as the anthelmintic also stimulates detectable calcium signals in the intestine of the gastrointestinal parasite *Ascaris suum*, through the *Asu*-TRP-2 orthologue^13^.

In this study, we transfected the mammalian Human Embryonic Kidney 293 cells with the *Brugia malayi* TRP-2b channel to determine if diethylcarbamazine still activates the parasite channel in a heterologous system. Here, by utilizing calcium imaging we demonstrate that application of diethylcarbamazine stimulates calcium signals in HEK293 cells transfected with *Bma-trp-2b*. The diethylcarbamazine mediated calcium signal can be inhibited using the TRPC antagonist SKF96365, as it does in both *Brugia malayi* and *Ascaris suum* preparations^7,8,13^. Additionally, the *Bma*-TRP-2b receptor can also be activated by arachidonic acid, an endogenous fatty acid, whose metabolites have multiple functions, including the activation of TRP channels^7,8,13,14^. We also observed that diethylcarbamazine stimulates weaker and less frequent detectable calcium signals in non-transfected HEK293 cells. These signals are also inhibited by SKF96365, suggesting that the endogenous mammalian TRPC channels are also sensitive to diethylcarbamazine, but less so, than the *Brugia malayi* TRP-2b channel. The activating effects on parasite and host TRP channels may explain diethylcarbamazine’s spastic effects on microfilaria and the stimulation of host immunity.

## Results

### TRPC expression in HEK293 cells

Previously, we have demonstrated that diethylcarbamazine stimulates robust calcium signals in the muscles of the filarial parasite *Brugia malayi* and intestine of the gastrointestinal parasite *Ascaris suum* via Transient Receptor Potential (TRP) channels^7,8,13^. We identified that these signals were mediated mostly by the TRPC orthologue, TRP-2, and some small part by the TRPM orthologues GON-2 and CED-11^7,8,13^. Consequently, we transfected full-length *Bma-trp-2b* (*Bm5246b.1*) into HEK293 cells to determine if the *Brugia malayi* TRP-2 channels were required for the response to diethylcarbamazine.

**Figure 1:**
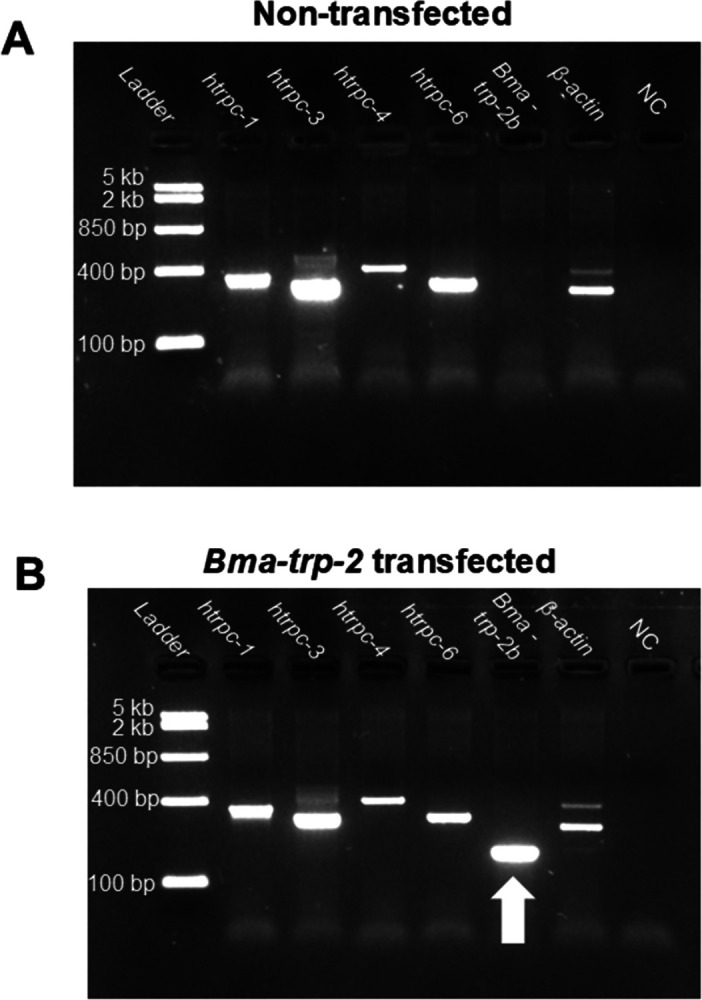
Presence of endogenous TRPC channels and detection of *Bma-trp-2b* in HEK293 cells. **A)** RT-PCR analysis of endogenously expressed TRPC channels (*htrp-1, htrp-3, htrp-4 & htrp-6)* in non-transfected HEK293 cells. Human *β-actin* was used as a positive control. N.C. = negative control, no cDNA template present. Ladder = FastRuler Middle Range DNA Ladder. Note the absence of the *Bma-trp-2b* band. **B)** RT-PCR analysis of *Bma-trp-2b*and endogenously expressed TRPC channels in transfected HEK293 cells. The arrow shows the presence of the *Bma-trp-2b* band. Original uncropped gel images are provided online (See Supplementary Fig. 1).

TRP channels are a superfamily of ion channels found in both invertebrates and vertebrates that play crucial roles in diverse physiological processes, and their opening is modulated by complex intracellular mechanisms including intracellular calcium, arachidonic acid or its metabolites, diacylglycerol, PIP2, ROS, ankyrin repeats and binding to CRIB sites^14^. HEK293 cells have been previously reported to express endogenous TRPC1, TRPC3, TRPC4 and TRPC6 channels^15,16^, but do not express TRPC2 or TRPC5^15^. We screened the cDNA pool generated from RNA extracted from 7-day old HEK293 cell populations using RT-PCR. We detected robust expression of all four TRPC channels in the HEK293 cells, Fig. 1A, as well as β-actin which was used as a control gene.

**Figure 2:**
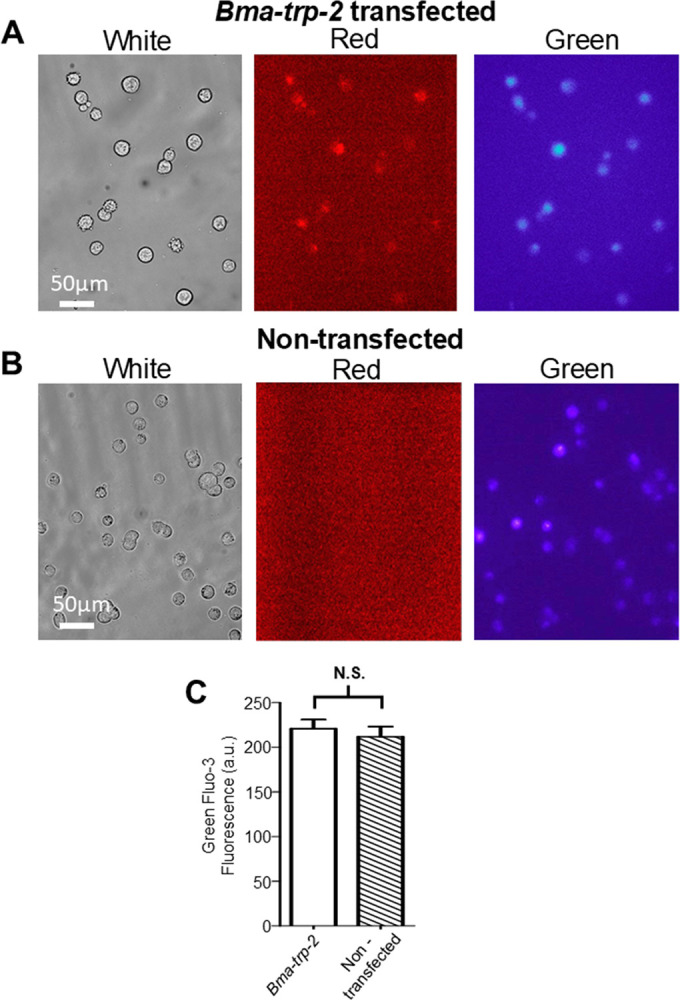
Fluorescence in HEK293 cells. **A)** Photomicrographs of HEK293 cells transfected with *Bma-trp-2b* after Fluo-3AM treatment under white light (*left panel)*, under green light (*middle panel)* and under blue light *(right panel*). Note the red fluorescence indicating expression of mCardinal found on the plasmid. Red cells were selected for recording with the assumption that the *Bma*-TRP-2b channel was also expressed in the cells. The Fluo-3AM fluorescence is under pseudo color settings which was used to measure the change in Ca^2+^ fluorescence. **B)** Photomicrographs of non-transfected HEK293 cells after Fluo-3AM treatment under white light (*left panel)*, under green light (*middle panel)* and under blue light *(right panel*). No red fluorescence was detected in non-transfected HEK293 cells (*middle panel)*. **C)** Average baseline fluorescence of Fluo-3 for every cell used from all recordings in this study for *Bma-trp-2b* transfected (white bar) and non-transfected (hatched bar) during the first minute of recording before any stimulus was applied. No significant difference was seen between the two cell populations. N.S. = not significant to *Bma-trp-2b* (*Bma-trp-2b* vs non-transfected, *P* = 0.5578 *t* = 0.5887, *df* = 76, unpaired *t*-test). *Bma-trp-2b n* = 1392 cells from 41 recordings, non-transfected *n* = 1576 cells from 37 recordings. All values represented as means ± SEM.

To determine if our *Brugia malayi* TRP-2b channel was being transcribed from our plasmid and if the presence of *Bma-trp-2b* affects the expression of the endogenous TRPC channels, we screened for the presence of all five channels from cDNA generated from the RNA extracted from transfected HEK293 cells. We observed similar expression of all four endogenous TRPC channels compared to the non-transfected cDNA pool as well as the presence of the *Bma-trp-2b* channel, Fig. 1B. Cells transfected with the mCardinal tagged *Brugia malayi* TRP-2b showed red fluorescence suggesting that our channel is being expressed in our system, Fig. 2A. We saw no red fluorescence in the non-transfected HEK293 cells, Fig. 2B.

### Diethylcarbamazine stimulates Ca signals in Bma-trp-2b transfected cells

With evidence that *Bma-trp-2b* is expressed in transfected HEK293 cells we wanted to determine if diethylcarbamazine could stimulate calcium signals via the parasite TRP-2b channel. All cells were initially visualized under white light to establish that the cells were healthy for recording, Fig. 2A (*left panel*). To identify which cells were suitable for recording and analyzing, populations were visualized under green light and any cell that had red fluorescence due to the expression of mCardinal found on the *Bma-trp-2b* plasmid, Fig. 2A (*middle panel*), was selected for observation and analysis. We then switched to the blue channel to record calcium signals and to verify that the selected cells with mCardinal fluorescence also had adequate Fluo-3AM loading, Fig. 2A (*right panel*).

**Figure 3:**
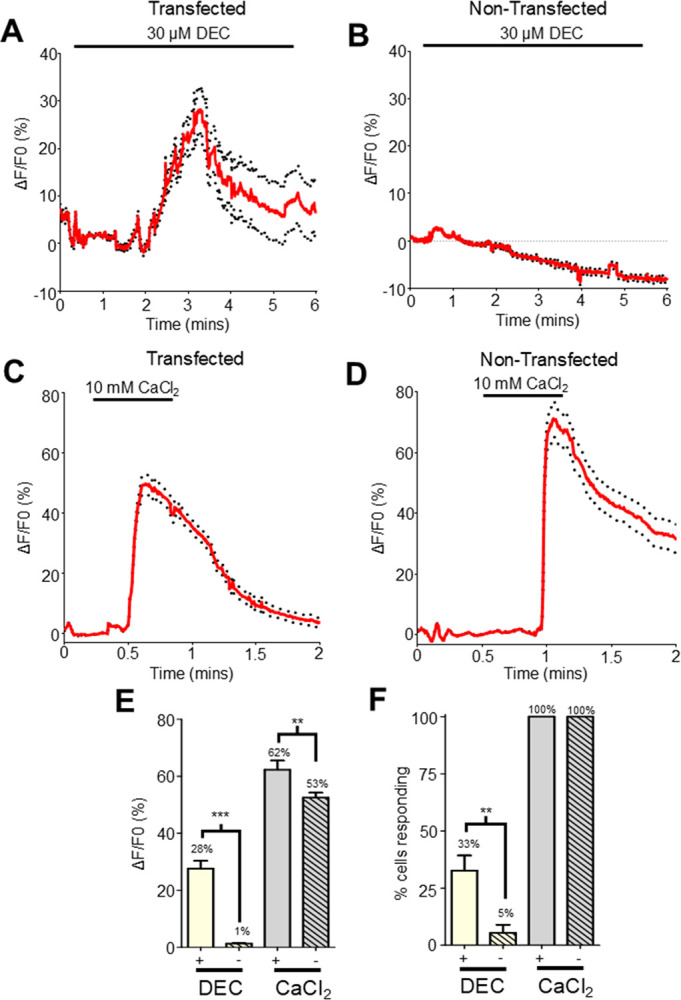
DEC increases the Ca^2+^ signal in *Bma-trp-2b* transfected HEK293 cells. **A)** Representative Ca^2+^ to 30 μM diethylcarbamazine (DEC) over five minutes in *Bma-trp-2b* transfected HEK293 cells. Horizontal bar indicates the DEC application. Red lines indicate average fluorescence; black dotted lines represent the ±SEM for all traces. **B)** Representative Ca^2+^ response to 30 μM DEC application in non-transfected HEK293 cells. Horizontal bar indicates the DEC application. **C)** Representative Ca^2+^ response to 10 mM CaCl_2_ in *Bma-trp-2b* transfected HEK293 cells. Horizontal bar indicates 10 mM CaCl_2_ application. **D)** Representative Ca^2+^ response to 10 mM CaCl_2_ in non-transfected HEK293 cells. Horizontal bar indicates 10 mM CaCl_2_ application. **E)** Total Ca^2+^ amplitudes in response to 30 μM DEC (yellow; + = 27.7% ± 2.7%, - = 1.3% ± 0.1%) and 10 mM CaCl_2_ (grey; + = 62.3% ± 3.2%, - = 52.5% ± 1.7%) in *Bma-trp-2b* transfected (+; clear) and non-transfected (-; hatched) HEK293 cells. *** significantly different: *Bma-trp-2b* DEC vs non-transfected DEC, *P* < *0.0001, t* = 16.78 *df* = 364, unpaired *t*-test. ** significantly different: *Bma-trp-2b* CaCl_2_ vs non-transfected CaCl_2_ *P 0.007, t* = 2.7 *df* = 547, unpaired *t*-test. *Bma-trp-2b n* = 6 recordings, DEC = 90/273 cells, CaCl_2_ = 273/273 cells. Non-transfected *n* = 7 recordings, DEC = 27/276 cells, CaCl_2_ = 276/276 cells. All values represented as means ± SEM. **F)** Mean percent of cells showing a Ca^2+^ response larger than the average spontaneous amplitudes in response to DEC (yellow; + = 32.7 ± 6.6%, - = 5.43 ± 3.53%) and 10 mM CaCl_2_ (grey; + = 100%, - = 100%) in *Bma-trp-2b* transfected (+; clear) and non-transfected (-; hatched) HEK293 cells. Note 100% of cells analyzed responded to 10 mM CaCl_2_. ** significantly different: *Bma-trp-2b* DEC vs non-transfected DEC *P 0.003, t* = 3.796 *df* = 11, unpaired *t*-test. *Bma-trp-2b n* = 6 recordings. Non-transfected, *n* = 7 recordings. All values represented as means ± SEM.

Following previous procedures, we applied 30 μM diethylcarbamazine for five minutes and observed large calcium signals, with an average peak amplitude of 28%, Fig. 3A & E. However, only 33% of cells tested provided a detectable calcium signal response, Fig. 3F. Initially this was surprising, given that all our selected cells showed mCardinal expression. We will address this question later in our manuscript. However, we still saw strong increases in fluorescence in response to our control 10 mM CaCl_2_ stimulus in 100% of cells tested, Figs. 3C, E & F.

As a further test of the calcium effect of diethylcarbamazine being mediated by *Bma*-TRP-2b, we also exposed non-transfected HEK293 cells to 30 μM diethylcarbamazine for five minutes. We observed that diethylcarbamazine did not stimulate a Ca^2+^ signal in non-transfected HEK293 cells, with the average maximal amplitude being 1%, which is lower than the 3% signal observed in non-stimulated cells Figs. 3B & E. We only detected a signal ≥ 3% in a small number of non-transfected cells (5%) in response to diethylcarbamazine Fig. 3F. The non-transfected cells did show robust increases to the 10 mM CaCl_2_ control stimulus like the transfected cells, Figs. 3D, E & F, confirming that these cells were healthy.

These results demonstrate that diethylcarbamazine stimulates an increase in calcium fluorescence in *Bma*-*trp-2b* transfected HEK293 cells while having a limited effect on the non-transfected cells and showing that diethylcarbamazine signals are dependent on the parasite TRP-2b channels.

### Carbachol enhances diethylcarbamazine signals

While application of 30 μM diethylcarbamazine was able to stimulate detectable calcium signals in *Bma-trp-2b* transfected HEK293 cells, only one third of the cells showed a response. One explanation is that opening of TRP-2 channels is modulated by other stimuli in addition to diethylcarbamazine that vary with the individual HEK cell. Activity of mammalian TRPC channels is complex and can be modulated by different signaling and scaffolding proteins which generate calcium microdomains that determine channel activity, clustering, and signal amplification^17^. The low percentage of cells responding to diethylcarbamazine in the transfected HEK293 cells (33%) may in part be due to lower intracellular calcium levels affecting the microdomains around the channels.

To explore the possibility of involvement of other modulatory stimuli, we pre-treated cells with 10 μM carbachol before the application of diethylcarbamazine. In HEK293 cells, carbachol signals via Gα_q_-coupled muscarinic acetylcholine receptors (mAChR) which activate PLC-β causing PIP_2_ hydrolysis, robust IP_3_, DAG production and an increase in intracellular calcium^18^. The increased intracellular calcium can replenish the calcium microdomains and increase activity of both the endogenous TRPC channels and the *Brugia malayi* TRP-2b channel, as seen with the *C. elegans* TRP-2 expressed in HEK293 cells^16,17,19^.

**Figure 4:**
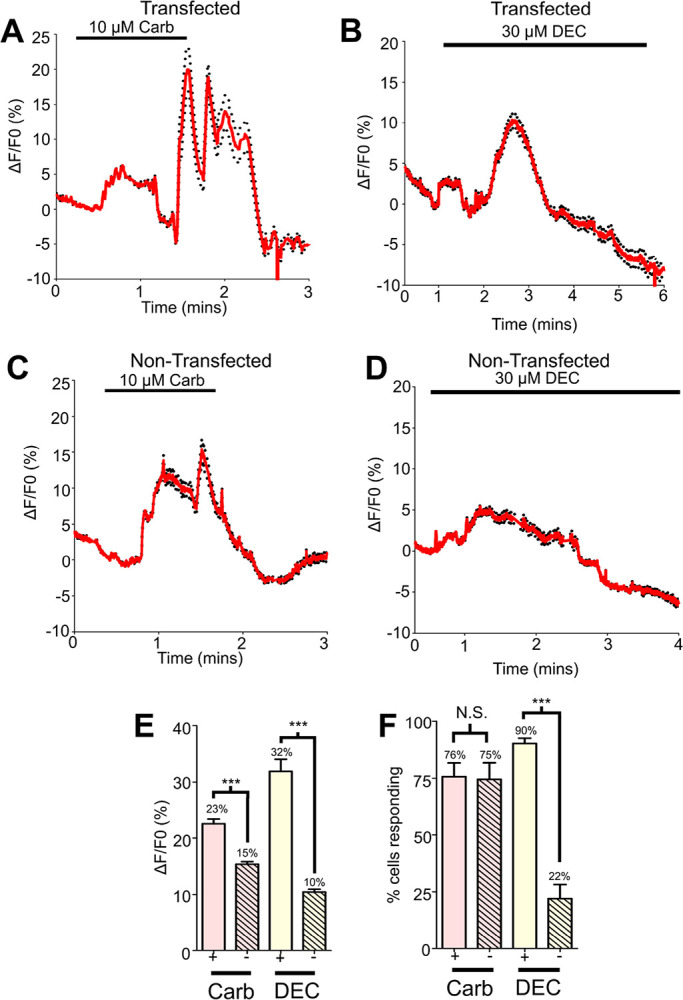
Carbachol pre-treatment increases diethylcarbamazine responses **A)** Representative Ca^2+^ response to 10 μM carbachol (carb) in *Bma-trp-2b* transfected HEK293 cells. Horizontal bar indicates carbachol application. Red lines indicate average fluorescence; black dotted lines represent ±SEM for all traces. **B)** Representative Ca^2+^ response to 30 μM diethylcarbamazine (DEC) in *Bma-trp-2b* transfected HEK293 cells after 10 μM carbachol. Horizontal bar indicates DEC application. **C)** Representative Ca^2+^ response to 10 μM carb in non-transfected HEK293. Horizontal bar indicates carbachol application. **D)** Representative Ca^2+^ response to 30 μM DEC in non-transfected HEK293 after 10 μM carbachol. Horizontal bar indicates DEC application. **E)** Total Ca^2+^ amplitudes in response to 10 μM carb (pink; + = 22.6% ± 0.8%, - = 15.4% ± 0.4%) and 30 μM DEC (yellow; + = 31.9% ± 2.2%, - = 10.4% ± 0.5%) in *Bma-trp-2b* transfected (+; clear) and non-transfected (-; hatched) HEK293 cells. *** significantly different to *Bma-trp-2b* carb (*Bma-trp-2b* carb vs non-transfected carb *P* = <0.0001*, t* = 8.115 *df* = 749, unpaired *t*-test). *** significantly different to *Bma-trp-2b* DEC (*Bma-trp-2b* DEC vs non-transfected DEC *P* = < *0.0001, t* = 5.467, *df* = 526, unpaired *t*-test). *Bma-trp-2b n* = 9 total recordings, carb = 336/449 cells, DEC = 405/449 cells. Non-transfected *n* = 10 total recordings, carb = 415/557 cells, DEC = 123/557 cells. All values represented as means ± SEM. **F**) Mean percent of cells showing a Ca^2+^ response larger than the average spontaneous amplitudes in response to 10 μM carb (pink; + = 75.7% ± 6%, - = 74.5% ± 7.3%) and to 30 μM DEC (yellow; + = 90.2% ± 2.3%, - = 22% ± 6.2%) in *Bma-trp-2b* transfected (+; clear) and non-transfected (-; hatched) HEK293 cells. 100% of cells analyzed responded to 10 mM CaCl_2_. N.S. not significantly different to *Bma-trp-2b* carb (*Bma-trp-2b* carb vs non-transfected carb *P 0.9042, t* = 0.1121 *df* = 17, unpaired *t*-test). *** significantly different to *Bma-trp-2b* DEC (*Bma-trp-2b* DEC vs non-transfected DEC *P* < *0.0001, t* = 9.804, *df* = 17, unpaired *t*-test). *Bma-trp-2b n* = 9 recordings. Non-transfected *n* = 10 recordings. All values represented as means ± SEM.

Application of 10 μM carbachol to *Bma-trp-2b* transfected cells generated distinct and sometimes oscillating increases in fluorescence, Fig. 4A with an average peak amplitude of 23% in 76% of cells tested, Figs. 4E & F. After the carbachol pre-treatment, cells were exposed to 30 μM diethylcarbamazine for five minutes resulting in a calcium signal profile, Fig. 4B, that had similar peak amplitudes (32%) as cells that were not pre-exposed to carbachol (compare Fig. 3E and Fig. 4E). However, pre-treatment with carbachol resulted in a larger percentage of cells showing detectable calcium responses, up from 33–90% of cells (Fig. 3F vs Fig. 4F). We observed large increases in fluorescence in response to our control 10 mM CaCl_2_ stimulus (see Supplementary Figs. 2A & C online). The presence of calcium microdomains appears to be necessary to increase the sensitivity of the *Bma*-TRP-2b channels in the HEK293 cell system in a similar manner to the *C. elegans* TRP-2 channel^19^.

With the transfected cells showing increased sensitivity to diethylcarbamazine after carbachol pre-treatment, we wanted to determine if non-transfected HEK293 cells also show increased sensitivity after carbachol pre-exposure. Again, carbachol generated characteristic calcium profiles with oscillations, Fig. 4C, and an average peak amplitude of 15%, which was significantly weaker than the transfected population, Fig. 4E, but the number of cells responding was not significantly different, 75% Fig. 4F. Interestingly, after the 10μM carbachol pre-treatment, 30 μM diethylcarbamazine generated a small but detectable increase in calcium fluorescence in the non-transfected cells Fig. 4D; but the average peak amplitude was significantly weaker (10%) compared to the diethylcarbamazine signal generated in *Bma-trp-2b* transfected cells, Fig. 4E, and the percentage of the cell population responding was also significantly lower compared to the transfected cells, 22%, Fig. 4F. Again, we observed large changes in fluorescence to our control 10 mM CaCl_2_ stimulus in these cells (see Supplementary Figs. 2B & C online).

These experiments demonstrate that the *Bma*-TRP-2b channel is sensitive to diethylcarbamazine, but when expressed in HEK293 cells the sensitivity is increased after pre-treatment with carbachol, due to modulation by a number of intracellular signaling mechanisms that may include elevating the calcium microdomain around the channels. We also observe that HEK293 cells have increased sensitivity to diethylcarbamazine after pre-treatment with carbachol. Our observations suggests that mammalian TRPC channels present in the HEK293 cells may be less sensitive receptor targets of diethylcarbamazine.

### Arachidonic acid stimulates Bma-TRP-2b channels to promote calcium entry

Arachidonic acid is a long chain polyunsaturated fatty acid that plays many roles in physiological processes. In mammals, arachidonic acid is oxidized by lipoxygenases, cyclooxygenases or CYP450 cytochromes. In filarial nematodes, arachidonic acid is not synthesized *de novo* but can be readily taken up from the host or converted from linoleic acid endogenously^20,21^. Arachidonic acid is converted into active and/or inactive poly unsaturated fatty acids (PUFAs) via ω-hydroxylases, cytochrome P450s and epoxygenase enzymes^22,23^. We have previously demonstrated that arachidonic acid has similar effects on the inward current and calcium profile in *Brugia malayi* muscles^7,8^, and calcium signaling in the *Ascaris suum* intestine as diethylcarbamazine^13^, suggesting that arachidonic acid or its metabolites are responsible for the activation of the TRP-2 channel in nematode tissues. We applied 30 μM arachidonic acid to determine its effects on *Bma-trp-2b* transfected HEK293 cells.

**Figure 5:**
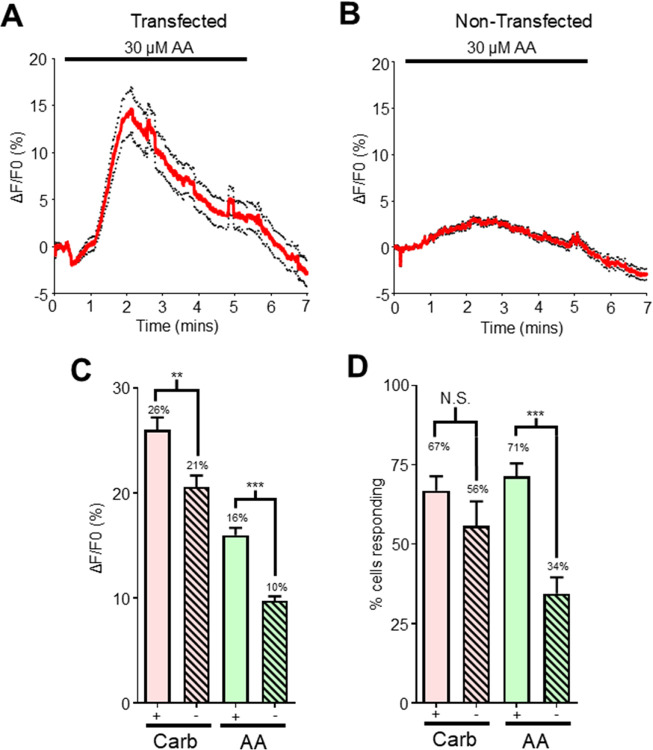
Arachidonic acid stimulates Ca^2+^ responses in Bma-trp-2b transfected HEK293 cells **A)** Representative Ca^2+^ response to 30 μM arachidonic acid (AA) after pre-treatment with carbachol in *Bma-trp-2b* transfected HEK293 cells. Black bar indicates AA application. Red lines indicate average fluorescence; black dotted lines represent the ±SEM for all traces. **B)** Representative Ca^2+^ response to 30 μM AA after pre-treatment with carbachol in non- transfected HEK293 cells. Black bar indicates AA application. **C)** Total Ca^2+^ amplitudes in response to 10 μM carbachol (pink; + = 26% ± 1.2%, - = 20.6% ± 1.1%) and to 30 μM AA (green; + = 16% ± 0.7%, - = 9.7% ± 0.4%) in *Bma-trp-2b* transfected (+; clear) and non-transfected (-; hatched) HEK293 cells. ** significantly different to *Bma-trp-2b* carbachol (*Bma-trp-2b* carbachol vs non-transfected carbachol *P 0.001, t* = 3.30 *df* = 529, unpaired *t*-test). *** significantly different to *Bma-trp-2b* AA (*Bma-trp-2b* AA vs non-transfected AA *P* < *0.0001, t* = 6.115, *df* = 454, unpaired *t*-test). *Bma-trp-2b n* = 14 total recordings, carbachol = 284/425 cells, arachidonic acid n = 303/425 cells. Non-transfected *n* = 10 total recordings, carbachol = 247/444 cells, arachidonic acid = 153/444 cells. All values represented as means ± SEM. **D)** Mean percent of cells showing a Ca^2+^ responses larger than the average spontaneous amplitudes in response to 10 μM carbachol (pink; + = 66.9% ± 4.5%, - = 55.7% ± 7.7%) and to 30 μM AA (green; + = 71.3% ± 4.1%, - = 34.4% ± 5.1%) in *Bma-trp-2b* transfected (+; clear) and non-transfected (-; hatched) HEK293 cells. N.S. not significantly different to *Bma-trp-2b* carbachol (*Bma-trp-2b* carbachol vs non-transfected carbachol, *P 0.1994, t* = 1.323 *df* = 22, unpaired *t*-test). *** significantly different to *Bma-trp-2b* AA (*Bma-trp-2b* AA vs non-transfected AA, *P* < *0.0001, t* = 5.641, *df* = 22, unpaired *t*-test). *Bma-trp-2b n* = 14 total recordings. Non-transfected *n* = 10 total recordings. All values represented as means ± SEM.

We pre-treated HEK cells with 10 μM carbachol and observed peak amplitudes of 26% Fig. 5C in 67% of cells recorded Fig. 5D. Following the pre-treatment with the carbachol, application of 30 μM arachidonic acid on *Bma-trp-2b* transfected HEK293 cells produced a calcium signal, Fig. 5A, that had an averaged peak amplitude of 16%, Fig. 5C, in 71% of cells tested, Fig. 5D. Application of the control 10 mM CaCl_2_ still resulted in large changes in fluorescence (see Supplementary Figs. S3 A & C online).

We also tested the effects of arachidonic acid on non-transfected cells. We repeated the same protocol with non-transfected cells and again saw that the carbachol calcium signal is significantly bigger in the transfected cells compared to the non-transfected cells, 26% vs 21% Fig. 5C; and similarly, we saw that the percentage of cells responding was not significantly different, 67% vs 56%, Fig. 5D.

Following application of 30 μM arachidonic acid, the peak calcium signal, Fig. 5B, was significantly smaller, 10% in the non-transfected HEK cells Fig. 5C; and the total number of cells showing a response to arachidonic acid was significantly lower, with only 34% of cells showing a detectable signal, Fig. 5D. All non-transfected cells demonstrated robust calcium amplitudes in response to 10 mM CaCl_2_ (see Supplementary Figs. S3 B & C online).

We concluded that application of exogenous arachidonic acid stimulates an increase in the intracellular calcium signal in *Bma-trp-2b* transfected and non-transfected HEK293 cells. However, the presence of *Bma*-TRP-2b significantly increases the amplitude and probability that cells will respond to arachidonic acid compared to cells that do not express the parasite channel, a phenotype like the diethylcarbamazine response.

### SKF-96365 inhibits the DEC mediated calcium signals in HEK293 cells

With the detection of calcium signals following diethylcarbamazine treatment, and the signal being stronger with the presence of the *Bma*-TRP-2b, we wanted to further test if the signal was being mediated through the TRP-2b channel. We have previously demonstrated that the diethylcarbamazine mediated signal can be inhibited in both *Brugia malayi* muscles and *Ascaris suum* intestines using the TRPC specific antagonist SKF96365^7,8,13^.

**Figure 6:**
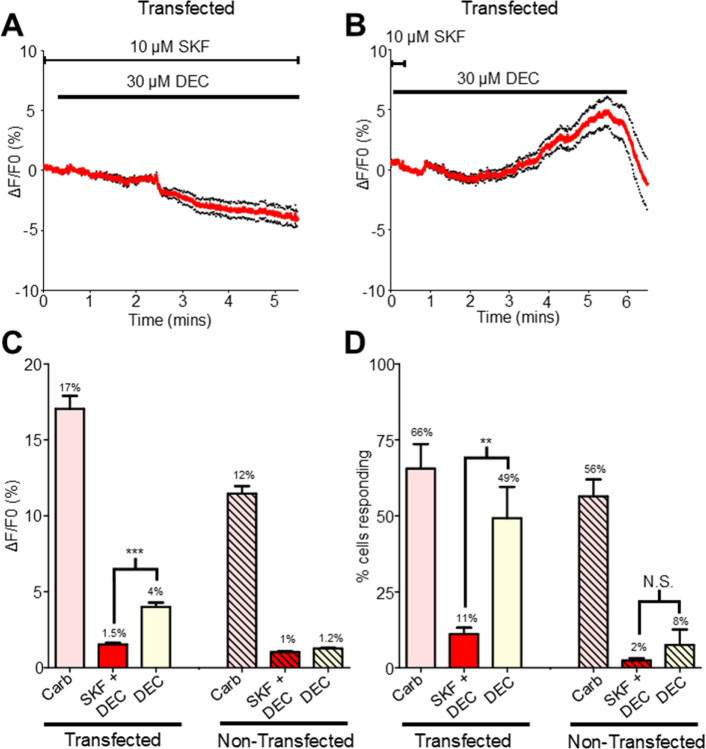
SKF96365 inhibits diethylcarbamazine signals in HEK293 cells **A)** Representative response to 10 μM SKF and 30 μM diethylcarbamazine (DEC) in *Bma-trp-2b* transfected HEK293 cells. Red lines indicate average fluorescence; black dotted lines represent ±SEM for all traces. **B)** Representative response to 30 μM DEC, black bar, after the removal of 10 μM SKF, short black bar in*Bma-trp-2b* transfected HEK293 cells. **C)** Total Ca^2+^ amplitudes in response to 10 μM carbachol (pink; transfected = 17.1% ± 0.9%, non-transfected 10.5% ± 0.5%), 10 μM SKF + 30 μM DEC (red; transfected = 1.5% ± 1.8%, non-transfected 1% ± 0.1%) and 30 μM DEC (yellow; transfected = 4% ± 0.3%, non-transfected 1.3% ± 0.1%) in *Bma-trp-2b* transfected (clear) and non-transfected (hatched) HEK293 cells. *** significantly different to SKF + DEC (*Bma-trp-2b* SKF + DEC vs *Bma-trp-2b* DEC *P* < *0.0001, t* = 8.929, *df* = 243, paired *t*-test). *Bma-trp-2b n* = 11 total recordings, carbachol = 164/244 cells, SKF + DEC = 27/244 cells, DEC = 120/244 cells. Non-transfected *n* = 10 total recordings carbachol = 164/293, SKF + DEC = 7/293 cells, DEC n = 22/293 cells. All values represented as means ± SEM. **D)** Mean percent of cells showing a Ca^2+^ response larger than the average spontaneous amplitudes in response to 10 μM carbachol (pink; transfected = 65.6% ± 8.1%, non-transfected 56.4% ± 5.6%), 10 μM SKF + 30 μM DEC (red; transfected = 11.1% ± 2.1%, non-transfected 2.4% ± 0.7%) and 30 μM DEC (yellow; transfected = 49.2% ± 10.3%, non-transfected 7.5% ± 5.1%) in *Bma-trp-2b* transfected (clear) and non-transfected (hatched) HEK293 cells. Note 100% of cells analyzed responded to 10 mM CaCl_2_. ** significantly different to SKF + DEC (*Bma-trp-2b* SKF + DEC vs *Bma-trp-2b* DEC *P* = 0.0055, *t* = 3.527, *df* = 10, paired *t*-test). N.S. not significantly different to non-transfected SKF + DEC (non-transfected SKF + DEC vs non-transfected DEC *P* = 0.326*, t* = 1.039, *df* = 9, paired *t*-test). *Bma-trp-2b n* = 11 total recordings. Non-transfected *n* = 10 total recordings. All values represented as means ± SEM.

After pre-treating the *Bma-trp-2b* transfected cells with 10 μM carbachol, we applied 10 μM SKF96365 for three minutes to allow the antagonist to inhibit the TRP channels. Application of the SKF96365 produced no significant changes in the calcium signal (see Supplementary Figs. S4 A & G online). Addition of 30 μM diethylcarbamazine in the presence of SKF96365 produced little or no change in the calcium signal, with an average maximal amplitude of 1.5%, Figs. 6A, C & D, indicating that the diethylcarbamazine mediated signal was inhibited. The SKF96365 was then washed, and the HEK cells were exposed to 30 μM diethylcarbamazine for an additional 5 minutes. The diethylcarbamazine produced a modest recovery of the calcium signal, Fig. 6B, with a mean increase in fluorescence of 4% in 49% of cells, Figs. 6C & D. Addition of 10 mM CaCl_2_ at the end of the experiment resulted in robust increases in all HEK293 cells (see Supplementary Figs. S4 E & G online). The inhibitory effects of SKF96365 support the hypothesis that the diethylcarbamazine signal in the *Bma-trp-2b* transfected cells is dependent on the activation of the *Brugia malayi* TRP-2b channel.

We also exposed non-transfected HEK293 cells to SKF96365, to determine if the observed increase in calcium upon diethylcarbamazine application was due to the activation of endogenous TRPC channels. We subjected non-transfected cells to the same protocol as described above and detected no increases in calcium in response to 10 μM SKF96365 alone or to 10 μM SKF96365 + 30 μM diethylcarbamazine, Figs. 6C & D (see Supplementary Figs. S4 B, C & G online). Interestingly, removal of SKF96365 and exposing cells to diethylcarbamazine alone did not increase the calcium signal, Figs. 6C & D (see Supplementary Fig. S4 D online). Application of 10 mM CaCl_2_ generated robust increases in fluorescence, indicating that the cells were healthy, and that the calcium probe was functional, (see Supplementary Figs. S4 F & G online). The inhibitory effects of SKF96365 on non-transfected HEK293 cell responses to diethylcarbamazine suggests that the detectable small changes in calcium fluorescence were due to activation of endogenous TRPC channels by diethylcarbamazine.

## DISCUSSION

### Diethylcarbamazine activates parasitic TRP-2 channels paralyzing filaria

Diethylcarbamazine is a classic anthelmintic that has been used for the treatment of lymphatic filariasis since 1947. Previously, it was understood that diethylcarbamazine’s mode of action is through the stimulation of the host’s immune system to clear microfilariae from the bloodstream and that it had no effects on parasites^3–6^. However, recent studies have demonstrated that diethylcarbamazine has a direct effect on adult parasites by generating temporary spastic paralysis^7^. This paralysis is mediated by diethylcarbamazine’s actions on TRP-2, a TRPC channel orthologue of *Brugia malayi*, and in some part through the TRPM-like channels, GON-2 and CED-11^7,8^. Opening of these parasite TRP channels leads to increases in cytosolic calcium in muscles and in turn activation of the SLO-1 K channels^7,8^. The paralytic effect of diethylcarbamazine can be potentiated by combining it with the newer anthelmintic, emodepside (activates SLO-1s), leading to further increases in calcium fluorescence in the muscle^8,12^. Once the microfilaria or adults are paralyzed, they are swept along and accumulate in the central vascular system of the liver, lymph nodes and lungs to become targets of the host immune system. The effects of diethylcarbamazine on the TRP-2 channels is also seen in the intestine of the gastrointestinal parasite *Ascaris suum*, showing that diethylcarbamazine has effects on TRP channels of other parasitic nematodes^13,24^.

#### Carbachol affects diethylcarbamazine calcium signals in TRP-2 transfected HEK293 cells:

**Figure 7:**
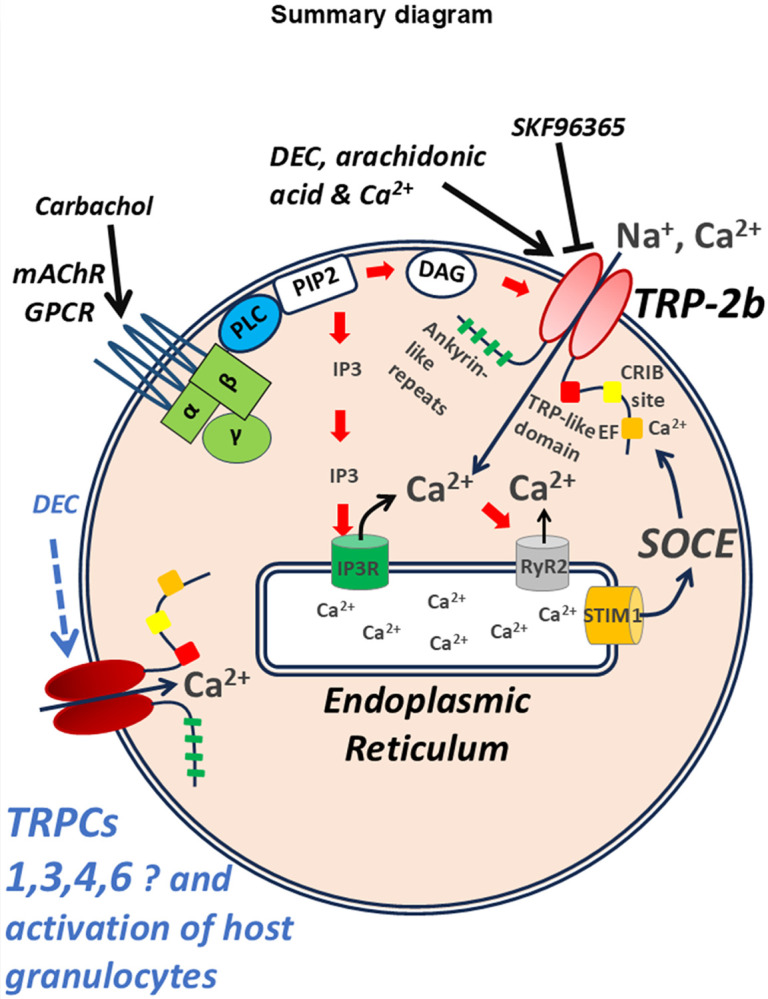
Summary diagram of the proposed mode of action of diethylcarbamazine (DEC) The *Brugia malayi* TRP-2 channel is permeable to Na^+^ and Ca^2+^ as a non-selective cation channel. The channel has ankyrin repeats at the N-terminal region and a TRP-like domain, a CRIB site, and an EF site where it interacts with Ca^2+^. Carbachol stimulation of muscarinic receptors (mAChR GPCR) with α, β and γ subunits can activate phospholipase C (PLC) to act on phosphatidylinositol 4,5 bisphosphate (PIP2) releasing diacylglycerol (DAG) and inositol trisphosphate (IP3). TRP-2 channels may be activated by diethylcarbamazine (DEC), arachidonic acid or its metabolites, PIP2, intracellular calcium, and are inhibited by the antagonist SKF96365. The release of Ca^2+^ from the endoplasmic reticulum into the cytoplasm may be due to activation of IP3 receptors (IP3R), or ryanodine receptors (RyR2). Reduced endoplasmic reticulum may activate STIM1 and store operated calcium entry (SOCE) through the TRP-2 channels.

Exposing transfected HEK293 cells to diethylcarbamazine led to increases in calcium and this stimulation was absent in non-transfected cells. Although diethylcarbamazine did stimulate calcium responses, the overall percentage of the HEK293 cell population that showed responses was surprisingly low. This low response rate may have been due to low intracellular calcium microdomains around the TRPC-like channels^17^. These calcium microdomains are modulated by numerous signaling molecules and cascades including: 1) GPCR signaling with activation of muscarinic receptors by carbachol; 2) intracellular calcium released from the endoplasmic reticulum into the cytoplasm through activation of IP3 receptors (IP3R) and ryanodine receptors (RyR2); or 3) reduction of calcium in the endoplasmic reticulum activating STIM1 and store operated calcium entry (SOCE), Fig. 7. The increase in intracellular calcium restores microdomains around the TRP channels with calcium interacting with: 1) the ankyrin repeats at the N-terminal region; 2) the TRP-like domains; 3) the CRIB site or; 4) an EF site, Fig. 7, allowing activation of the channel by diethylcarbamazine. The increase in the number of cells activated by diethylcarbamazine following carbachol treatment showed that the low number of responses without prior carbachol treatment was not due to unsuccessful transfection and translocation of the TRP-2 channel to the plasma membrane.

### Arachidonic acid and activation of TRP-2 channels

Arachidonic acid is an important polyunsaturated fatty acid that is involved in many signaling roles. In mammals, metabolism of arachidonic acid by lipoxygenases, cyclooxygenases or CYP450 cytochromes generates metabolites that are involved in inflammation, pain and immune responses^25,26^. The effects of diethylcarbamazine in mammals is reported to include effects on metabolism of arachidonic acid^4^. During filarial parasitic infections, parasites readily take up arachidonic acid from the host as they are unable to synthesize the compound *de novo*^20,21^. Arachidonic acid is then converted into active and/or inactive poly unsaturated fatty acids (PUFAs) via ω-hydroxylases, cytochrome P450s and epoxygenase enzymes by the parasite^22,23^. Arachidonic acid, like diethylcarbamazine, activates TRP channels in *Brugia malayi* producing in their muscle cells an inward current and an increase in cytosolic calcium.^7,8^, as it does in the *Ascaris suum* intestine^13^. Arachidonic acid or its metabolites activate nematode TRP channels.

We observed exposure of the *Bma-trp-2b* transfected HEK293 cells to arachidonic acid, generated detectable and frequent calcium signals that mimicked diethylcarbamazine. In non-transfected cells, we also observed an increase in calcium, but the signal was weaker and seen in fewer cells but nonetheless mimicked the diethylcarbamazine profile seen in transfected cells. Arachidonic acid, or its metabolites, have effects on the parasite TRP-2 but also have effects on mammalian TRP channels as well.

### Activation of host immune cells by diethylcarbamazine and TRPC channels

Diethylcarbamazine somehow activates host immune responses and inhibits host and filarial cyclooxygenase and host leukotriene synthases^5,27,28^. This inhibition leads to a reduction of PGE2, PGD2 and PGI2 prostaglandins that is associated with clearing of the microfilaria from the bloodstream produced by microfilarial adhesion to endothelial cells, eosinophils, platelets, and neutrophils^29,30^. The mechanism by which diethylcarbamazine activates the immune cells and inhibits these pathways has not been explained.

In this study we demonstrated that the presence of *trpc1, trpc3, trpc4* and *trpc6* message in HEK293 cells and that diethylcarbamazine can stimulate small calcium signals in non-transfected HEK293 cells. Although the diethylcarbamazine signal was weaker in amplitude and was less frequent compared to cells transfected with the *Bma-trp-2b*, the diethylcarbamazine signal was inhibited using the TRPC specific antagonist SKF96365. These observations suggest that diethylcarbamazine can activate mammalian cells through the stimulation of their TRPC channels and increases in intracellular calcium.

### Combined effects of diethylcarbamazine on filariaTRP-2 and host immune cell TRPCs

We suggest that diethylcarbamazine, in addition to activation of TRP-2 channels, can activate TRPC channels of host immune cells including granulocytes and macrophages, Fig. 7, like the endogenous TRPC channels of HEK293 cells. Family members of different TRP channels are present and functional on multiple types of immune cells. TRPC channels have been identified on macrophages, neutrophils and T lymphocytes^31^. Activation of their TRPC channels leads to pro-inflammatory effects, increasing levels of cytokines that stimulate, recruit and amplify the immune response to clear pathogens^31^. TRPC channels also modulate the level of chemokines and cytokines with chemotactic activities from macrophages that regulate localization and migration of lymphocytes and dendritic cells^31,32^. Interestingly, during helminth induced diseases, macrophage activation is suppressed by impairing the STIM1-TRPC1 complex with reduced store operated calcium release (SOCE) and calcium entry. The activation of immune cells via TRPC channels is an important mechanism for stimulating inflammatory pathways and for clearing helminth parasites^33^.

## Conclusion

We have demonstrated that diethylcarbamazine action is dependent on the *Brugia malayi* TRP-2b channel that leads to increases in cytosolic calcium in transfected HEK293 cells. Increasing the frequency of diethylcarbamazine mediated calcium responses in *Bma-trp-2b* transfected HEK293 cells is contingent on pre-treatment with carbachol, due to a number of intracellular signaling mechanisms that include elevating the calcium microdomain around the channels. The diethylcarbamazine signal is inhibited by the TRPC specific inhibitor SKF96365, further supporting a role for diethylcarbamazine acting on the parasite TRP-2 channel. Lastly, we have established that diethylcarbamazine can stimulate weaker and less frequent calcium signals in non-transfected HEK293 cells, which are also inhibited by SKF96365. These results suggest that mammalian TRPC channels are also diethylcarbamazine targets that could lead to the stimulation of immune responses against the parasite.

## Materials and Methods

### HEK283 maintenance

The Human Embryonic Kidney 293 (HEK293) cell line was used for all experiments in this study. HEK293 cells were maintained in a humidified growth incubator set to 37°C in 5% CO_2_ following previous published methods^34^. Cells were cultured in 10 mL Dulbecco’s Modified Eagle Medium (DMEM 1X) supplemented with 10% (v/v) Fetal Bovine Serum (FBS) and 1% (v/v) Penicillin-Streptomycin (Pen/Strep). Media was exchanged every 3 days. Cells were passaged for a maximum of 14 generations. Passaging was achieved by discarding the previous media and washing cells with 1X PBS warmed to 37°C to remove cellular debris. Cells were treated with 2.5 mL of 0.25% trypsin-EDTA warmed to 37°C and incubated at 37°C in 5% CO_2_ for 2–3 minutes to detach cells. After incubation, 10 mL of warmed DMEM + 10% FBS + 1% Pen/Strep, was added as the serum contains deactivators of trypsin. Cells were gently pipetted up and down to break clumps and to help detach cells remaining on the plate. 1 mL of cells were added to 37°C warmed 10 mL fresh DMEM + 10% FBS + 1% Pen/Strep in new plates and placed in the incubator. Cells were frozen during every passage by adding 950 μL cell media to 50 μL 5% (v/v) DMSO and mixing gently before being stored at −80°C.

### Brugia supply and maintenance

Live adult female *Brugia malayi* were shipped overnight from the NIH/NIAID Filariasis Research Reagent Resource Centre (FR3; College of Veterinary Medicine, University of Georgia, Athens, USA). *Brugia malayi* were maintained in non-phenol red Hyclone Roswell Park Memorial Institute (RPMI) 1640 media containing 10% heat-inactivated FBS and 1% penicillin-streptomycin. Parasites were separated individually into a 24 well microtiter plate containing 2 mL of the RPMI media. Parasites were held in an incubator set to 37°C in 5% CO_2_.

### Cloning and maintenance of Bma-trp-2b

Adult *Brugia malayi* worms were snap-frozen in liquid nitrogen and crushed into fine powder in a 1.5 mL micro-centrifuge tube using Kimble Kontes Pellet Pestle (Fisher Scientific, Waltham, MA, USA). Total RNA was extracted using TRIzol Reagent (Life Technologies, Carlsbad, CA, USA) according to manufacturer’s instructions. cDNA was synthesized using SuperScript VILO Master Mix (Life Technologies, Carlsbad, CA, USA) from approximately 1 μg RNA as template. Full-length *Bma-trp-2b* (*Bm5246b.1*) was amplified using Platinum SuperFi Polymerase Master Mix (Thermo Fisher, Waltham, MA, USA) using the primers SSK160F and SSK160R (Table 1). Primers were made with the sequences flanking the expression vector pTarget (P2A::mCardinal), with SSK160F flanking the BamHI site and SSK160R flanking KpnI and the P2A site. Sequencing of the *pTarget (P2A::mCardinal)::Bma-trp-2b* plasmid is available in supplementary information (see Supplementary data online). The amplicon was analyzed using agarose gel electrophoresis and was purified using NucleoSpin Gel and PCR Clean-up kit (Macherey-Nagel, Düren, Germany). The purified *trp-2b* cDNA was cloned into the pTarget vector using Infusion HD Cloning Kit (Takara Bio, San Jose, CA, USA) under the manufacturer’s guidelines. Upon cloning, the plasmids were sequence-verified and ready for transfection into HEK293 cells.

The plasmid was added to Mix and Go! Competent JM109 *Escherichia coli* bacteria (Zymo Research, Irvine, CA, USA) which were grown in LB media with 25 μg/ml ampicillin at 37°C for 16–18 hours. The plasmid was purified from bacterial lysates using Monarch^®^ plasmid miniprep kit (New England Biolabs, Ipswich, MA, USA) and diluted in nuclease free water. The extracted plasmid concentrations were analyzed using an Implen NanoPhotometer^®^ N120 (Implen, Munich, Germany). All plasmid stocks were stored at −20°C.

### Transfection of HEK293 cells

For transfection, we followed techniques as previously described^34^. Briefly, 500 μL of HEK293 cells were cultured in 5 ml of DMEM + 10% FBS and 1% Pen/Strep for 72 hours or until ~ 90% confluence was achieved in a humidified growth incubator set to 37°C and 5% CO_2_. Transfection was achieved using cationic lipid-based reagent Lipofectamine^®^ 3000 transfection kit (Invitrogen, Thermo Fisher Scientific, Waltham, MA, USA) according to manufacturer’s instructions. Before transfection, old media was discarded, and cells were washed with 5 mL warm 1X PBS. Cells were incubated in OPTI-MEM^®^ I (1X) Reduced Serum Medium + GlutaMAX^™^ -I for 40 minutes at 37°C in 5% CO_2_. Whilst cells were incubating, the transfection media was prepared by adding 5–8 μg of plasmid to a ratio of 3.75 μL lipofectamine per μL plasmid in 125 μL OPTI-MEM and by adding 2 μL P3000 per μL of plasmid to 125 μL OPTI-MEM. All components were mixed and incubated for 15 minutes at room temperature in the dark to improve transfection quality. When added, the transfection solution was applied over the area of the cell plate and the sample was incubated for a minimum of 6 hours at 37°C in 5% CO_2_. After the transfection period the OPTI-MEM solution was discarded and replaced with DMEM supplemented with 10% FBS and cells were placed in a humidified growth incubator set to 30°C at 5% CO_2_ for 48 hours to promote expression but slow cell growth. Before experiments, cell health was assessed via light microscope.

### HEK293 cDNA synthesis and RT-PCR detection of TRP channels

To obtain cDNA we cultured HEK293 cells either transfected with 5–8 μg of the *Bma-trp-2b* expression vector or without the vector at 37°C at 5% CO_2_ for 7 days. HEK293 cells were homogenized in 1 mL of TRIzol by gentle pipetting, followed by total RNA extraction according to the TRIzol Reagent protocol. One microgram (1 μg) of total RNA from each culture was used to generate cDNA by reverse transcription (RT) using SuperScript VILO^™^ Master Mix following the manufacturer’s protocol. PCR was conducted to detect the presence of the known endogenous human TRPC channels TRPC1, TRPC3, TRPC4 and TRPC6 by targeting the cDNA of *htrp-1, htrp-3, htrp-4* and *htrp-6* using previously published primers that target the encoding regions of each gene^15^ (Table 1). To measure the expression of *Bma-trp-2b* cDNA, we screened using primers targeting the encoding region of *trp-2b* that we have previously used^7^ (Table 1). *hβ-actin* was used as a reference gene^15^. Negative controls included enzyme, water, and both forward and reverse primers for *hβ-actin* with no cDNA template. The cycling conditions for PCR were an initial denaturation for 2 mins at 95°C, followed by 35 cycles of 95°C for 30 secs, 61°C for 30 secs, 72°C for 20 secs, and a final extension at 72°C for 10 mins using GoTaq^®^ G2 Hot Start Green Master Mix (Promega, Madison, WI, USA). PCR products were then separated on a 2% Agarose gel containing SYBR^®^ Safe DNA Gel Stain, followed by visualization and images were captured using an Azure 600 imaging system set to SYBR Safe (Excitation 472nm, Emission 595nm).

### Capturing cell image:

White and red fluorescent images were captured using Occular 2.0.1.496 imaging software (Digital Optics, Auckland, New Zealand). For white light images the exposure settings were 10 ms with no binning Figs. 2A & B (*left panels*). Red fluorescence imaging was achieved using a Lambda LS Xenon bulb lightbox which delivered light via a fiber optic cable to the microscope (Sutter Instruments, Novato, CA, USA) which passed through a green/red filter box (EF-4 AT TRITC/CY3 LP; Excitation: 527.5–552.5nm, Emission 575nm Long Pass, Dichroic Mirror) (Chroma Technology, Bellows Falls, VT, USA). Fluorescent light emission was controlled by using the shutter. The exposure settings for the mCardinal fluorescent images were 500 ms with 2x binning Figs. 2A & B (*middle panels*). For Fluo-3 fluorescence, images were captured using MetaFluor 7.10.2 (MDS Analytical Technologies, Sunnyvale, CA) with exposure settings at 250 ms with 2x binning under pseudo-color settings to illustrate calcium levels Figs. 1A & B (*right panels*). All images were captured using a Photometrics Retiga R1 Camera (Teledyne Photometrics, Tucson, AZ, USA).

### Preparation and loading Fluo-3AM

Before recording, DMEM + 10% FBS media is discarded, and cells are washed with warmed 1X PBS to remove cellular debris. Cell detachment was achieved by adding 500 μL of 0.25% trypsin-EDTA and incubating at 37°C in 5% CO_2_ for 2–3 minutes. Next, 5 mL of DMEM + 10% FBS was added to deactivate the trypsin, and cells were resuspended by gentle pipetting. Clean 12 mm circle coverslips coated with poly-D-lysine following methods previously described^34^ were placed in a clean Petri dish and 20 μL of cell media is added to each disc. To promote cell adhesion, DMEM + 10% FBS was added to cover the coverslip, and samples are incubated for a minimum of 2 hours before recording at 37°C in 5% CO_2_.

After incubation the 12 mm glass cover slip was placed in a Warner RC26G laminar flow chamber (Warner Instruments, Holliston, MA, USA), immersed in HEK293 cell buffer (150mM NaCl, 5 mM KCl, 5 mM CaCl_2_, 1 mM MgCl_2_, 15 mM HEPES and 10 mM glucose, pH 7.3) and cells were visualized under white light on a Nikon Eclipse TE3000 microscope fitted with a 20X/0.45 Nikon PlanFluor objective to ensure that they appeared healthy before Fluo-3AM loading (Figs. 2A & B). Fluo-3AM (Sigma-Aldrich, St. Louis, MO, USA) loading was achieved by incubating the cells in HEK293 cell buffer containing 5 μM Fluo-3AM and 10% Pluronic F-127 (10% v/v) for 20 minutes with the recording chamber connected to a Dual Automatic Temperature Controller (Warner Instruments, Holliston, MA, USA) maintained at 36–37°C. After incubation, the Fluo-3AM solution was discarded, and the sample was incubated in HEK293 cell buffer for an additional 10 minutes at 36–37°C to promote calcium loading. Before performing calcium imaging, cell transfection of the *Bma-trp-2b* plasmid was verified under green light using a long pass green/red filter box. Transfected cells that were fluorescing red were selected for recording Fig. 2A. We observed no red fluorescence in non-transfected cells Fig. 2B. Fluo-3AM loading was confirmed under blue light using a blue/green filter box (EF-4 AT EGFP/FITC/CY2/ALEXA FLUOR Band Pass; Excitation: 465–495nm, Emission: 525–545nm, Dichroic Mirror) (Chroma Technology, Bellows Falls, VT, USA) and visualized under pseudo-color settings using MetaFluor 7.10.2 (Figs. 2A & B). Any cell that did not show Fluo-3 fluorescence regardless of fluorescence under the red channel was discarded. We observed no significant difference in the levels of Fluo-3 fluorescence between *Bma-trp-2b* transfected and non-transfected cells (Fig. 2C).

### Measurement of Ca^2+^ fluorescence

All recordings were performed on a Nikon Eclipse TE3000 microscope fitted with a 20X/0.45 Nikon PlanFluor objective, using a Photometrics Retiga R1 Camera. Light control was achieved using a Lambda 10 − 2 with a shutter controller. Fluorescence was achieved using a Lambda LS Xenon bulb lightbox (Sutter Instruments, Novato, CA, USA) which delivered light via a fiber optic cable to the microscope which passed through a blue/green filter box. Fluorescent light emission was controlled by using the shutter. Minimal illumination exposure was used to prevent photobleaching.

During recordings cells were continuously perfused with HEK293 cell buffer solutions. Cells were exposed to either 10 μM carbachol for one minute, 30 μM diethylcarbamazine (DEC) for five minutes, 30 μM arachidonic acid (AA) for five minutes, 10 μM SKF96365 for three minutes (SKF), 10 μM SKF96365 + 30 μM DEC (SKF + DEC) for 5 minutes or 10 mM CaCl_2,_ which was used as a positive control to determine cell viability. If a cell failed to respond to the 10 mM CaCl_2_ control signal it was discarded from the data pool regardless of previous signals to our compounds. Every experiment in this study has a 100% cell response to 10 mM CaCl_2_. A change in fluorescence ≥ 3% was classified as a positive response to a compound as the average spontaneous change in cells continuously perfused with HEK293 buffer was ~ 2.5%.

All solutions were delivered to the chamber under gravity feed through solenoid valves controlled using a VC-6 six-channel Valve Controller through an inline heater set at 37°C (Warner Instruments, Holliston, MA, USA), at a rate of 1.5mL/min. At the start of all experiments, cell samples were left under blue light for a minimum of 1 minute to promote settling and equilibration of the fluorescent signal and to monitor any cells that may detach from the coverslip before the application of compounds.

All calcium signal recordings were acquired and analyzed using MetaFluor 7.10.2 with exposure settings at 250 ms with 2x binning. Maximal percent calcium signal amplitudes (ΔF) were calculated using the equation F1-F0/F0 × 100, where F1 is the fluorescent value and F0 is the baseline value. All F0 values were taken at the time point each compound was applied for every cell analyzed. All representative response traces are presented as the average percent change in calcium fluorescence with the standard error of the mean (± SEM) of all cells from a single recording. Traces were generated by converting the calcium signal profiles of all the cells from a single recording to percentages using the previously described ΔF/F0 equation, with the time of stimulus application being F0 (0% for all cells) and 100% being the peak value for each cell. The mean percentage change in fluorescence and the SEM was calculated for each time point during the application of each compound. All baseline values were taken at the time point each compound was applied for every cell. Traces are represented as mean ± SEM.

### Statistical Analysis

Statistical analysis of all data was done using GraphPad Prism 9.0 (GraphPad Software, Inc., La Jolla, CA, USA). To ensure reproducibility, we repeated our experiments: the numbers of cells, number of preparations, the concentrations, and durations of applications are provided in the legends of the figures. Analysis of calcium amplitudes and percentage of cells responding was done using paired or unpaired student *t*-tests with *P* < *0.05* being considered significant. The data are presented as mean ± SEM for each treatment.

### Chemicals

Source of chemicals; SKF96365 was purchased from Tocris. Carbachol was purchased from EMD Millipore. All other chemicals were supplied by Sigma Aldrich.

## Supplementary Material

Supplementary Files

This is a list of supplementary files associated with this preprint. Click to download.

- Table1.docx
- pTargetP2AmCardinalBmatrp2bsequence.pdf
- SupplementaryFigures.pdf

## Data Availability

The data that supports the findings within the manuscript and its Supporting Information files are openly available at DOI: 10.5061/dryad.z34tmpgsx
